# Blood pressure control for optimal brain health: a systematic literature review

**DOI:** 10.1002/alz70860_105675

**Published:** 2025-12-23

**Authors:** Sara KamaliZonouzi, Melika Arab Bafrani, Vladimir Hachinski, Abolfazl Avan

**Affiliations:** ^1^ Tehran University of Medical Sciences, Tehran, Tehran, Iran (Islamic Republic of); ^2^ Weil institute of neuroscience, UCSF, San Francisco, CA, USA; ^3^ University of Western Ontario, London, ON, Canada

## Abstract

**Background:**

Hypertension, a leading modifiable risk factor for cognitive decline, negatively affects cerebral vasculature, particularly in regions critical for executive function. The relationship between blood pressure (BP) changes and brain (physical, mental, and social) health remains unclear.

**Method:**

This systematic review and meta‐analysis, adhering to PRISMA guidelines, explored the association between blood pressure control and brain health outcomes – specifically, cognitive and executive dysfunction, stroke, and post‐stroke dementia. PubMed and Embase were searched for randomized controlled trials (RCTs) and cohort studies published up to January 2025. Eligible studies included those assessing patients with or without interventions aimed at regular blood pressure control. Data extraction was performed independently by two reviewers, and the risk of bias in included studies was assessed using validated tools. A random‐effects model was employed to pool effect sizes. Heterogeneity was quantified using the *I* ^2^ statistic, while publication bias was examined with funnel plots and Egger's test.

**Result:**

After screening 5049 citations, 382 studies were selected for data extraction and analysis. Our preliminary analysis suggests that both high systolic blood pressure and low diastolic blood pressure may negatively impact brain health and cognitive performance. Ongoing analyses will examine the effects of blood pressure fluctuations across different life stages and explore the impact of antihypertensive drugs on mental well‐being, cognitive abilities, and cerebral imaging results.

**Conclusion:**

This systematic review and meta‐analysis underscore the potential benefit of targeted blood pressure interventions for improving cognitive function, mental health, and overall quality of life. These findings provide a basis for future research and emphasize the importance of developing strategies to optimize health‐related quality of life (HRQL) through effective blood pressure management.

